# Association between COVID-19 Infection and Miscarriages, What We Really Know?

**DOI:** 10.3390/diseases11040173

**Published:** 2023-11-29

**Authors:** Ioannis Chrysanthopoulos, Anastasios Potiris, Eirini Drakaki, Despoina Mavrogianni, Nikolaos Machairiotis, Paul Zarogoulidis, Theodoros Karampitsakos, Pavlos Machairoudias, Dionysios Vrachnis, Periklis Panagopoulos, Peter Drakakis, Sofoklis Stavros

**Affiliations:** 1 First Department of Obstetrics and Gynecology, Alexandra Hospital, Medical School, National and Kapodistrian University of Athens, 115 28 Athens, Greece; johnchrysan@med.uoa.gr (I.C.); eirinidrak@med.uoa.gr (E.D.); dmavrogianni@med.uoa.gr (D.M.); pdrakakis@med.uoa.gr (P.D.); 2 Third Department of Obstetrics and Gynecology, University General Hospital “ATTIKON”, Medical School, National and Kapodistrian University of Athens, 124 62 Athens, Greece; nikolaosmachairiotis@gmail.com (N.M.); theokarampitsakos@hotmail.com (T.K.); pavlosmach@gmail.com (P.M.); sfstavrou@med.uoa.gr (S.S.); 3 Pulmonary Department, General Clinic Euromedica, 544 54 Thessaloniki, Greece; pzarog@hotmail.com; 4 Medical School, National and Kapodistrian University of Athens, 115 27 Athens, Greece; dionisisvrachnis@gmail.com

**Keywords:** SARS-CoV-2, COVID-19, miscarriage, pregnancy loss, reproduction

## Abstract

Background: COVID-19 is a modern worldwide pandemic that affected and continues to affect millions of people around the world. Since the discovery that angiotensin-converting enzyme 2 (ACE2) is the binding site for COVID-19 to achieve cell entry, there has been a continuous debate about the effect of COVID-19 infection in first and second trimester abortions. The aim of this review is to investigate the impact of COVID-19 infection on the incidence of miscarriage. Furthermore, we seek to identify potential pathophysiological mechanisms of early pregnancy loss present in infected women. Methods: A literature review was conducted on different databases, including PubMed, Google Scholar, Ovid, Science Direct, Scopus, and Cochrane library, between 1 January 2020 and 31 August 2023. A total of 364 articles were identified and 32 articles were ultimately included in the review. Results: There are several case studies that provide evidence that early pregnancy loss is associated with COVID-19 infection. These findings are not further confirmed by the majority of systematic reviews and meta-analyses, which demonstrate that the total number of miscarriages do not differ significantly between infected and non-infected groups. Furthermore, there are also case reports that associate COVID-19 infection with late second trimester abortions. Conclusions: Given that the virus persists globally, it is important to gain a better understanding of its associated risks in the reproductive process, and larger, more homogeneous, and controlled studies are required to obtain more robust data that can be meta-analyzed to obtain an overview of this potential relationship.

## 1. Introduction

The World Health Organization (WHO) announced in December 2019 that a respiratory disease known as Severe Acute Respiratory Syndrome Corona Virus (SARS-CoV), named COVID-19 for convenience, had taken the form of a pandemic and was spreading globally [1]. The impact of COVID-19 infection in pregnancy initiated with the fact that infected pregnant women who were admitted to the hospital were more likely to require invasive ventilation or intensive care than show more subtle symptoms such as fever, cough, dyspnea, and myalgia [2]. Moreover, a relatively increased risk was noted as it was hypothesized that COVID-19 affects the granulosa cells and ovarian tissue, which impairs ovulation and ovarian function and increases the risk of miscarriage and infertility [3].

Miscarriage is defined as the unintended loss of a pregnancy prior to the 22nd week of gestation by the American College of Obstetrics and Gynecology (ACOG). According to worldwide demographics, an average of 10% to 26% of miscarriages occur before the 20th week of gestation [4,5,6]. Risk factors for severe COVID-19 infection in pregnancy were later described as comprising of non-white ethnicity, chronic hypertension, preexisting diabetes, a high maternal age, and a high body mass index. In a large-scale study, inflammation of the placenta during pregnancy was found to be one of the reasons for miscarriages. However, infants born from infected mothers did not have an increased risk of abortion, amniotic fluid abnormalities, cyanosis, or congenital defects [7].

In terms of COVID-19’s effect on reproductive potency, it has been established that angiotensin-converting enzyme 2 (ACE2) is a shared component of both the reproductive biological processes while serving as a binding site for COVID-19 to achieve cell entry. A gene on chromosome X encodes the sole catalytic domain of ACE2, a transmembrane zinc metallopeptidase. Greater interaction with ACE2 is possible because of the homogeneous distribution of S proteins on the viral surface. Cellular transmembrane serine protease 2 (TMPRSS2), which is present on the membrane of the host cell, facilitates virus entrance into the cell by activating the S protein after binding to ACE2 [8]. Consequently, it is anticipated that cells in many tissues and organs expressing the ACE2 receptor are susceptible to damage [9]. Numerous organs and tissues, such as the lungs, colon, kidney, testes, and many others, express the ACE2 receptor. Because these entry factors are expressed in organs, COVID-19 has a higher chance of spreading there. Effects on fertility and the potential of reproduction have previously been suspected because the ACE2 receptor is expressed in both the male and female reproductive systems [10,11]. The involvement of the human reproductive system demonstrates a possible infection in the primordial germ cells, causing a malfunction of the reproductive glands and a possible alteration in the gametes [12].

The aim of the present literature review is to investigate the impact of COVID-19 infection on the incidence of miscarriage. Furthermore, we seek to identify potential pathophysiological mechanisms of early pregnancy loss present in infected pregnant women.

## 2. Materials and Methods

A literature review was conducted with the aim to describe the relationship between COVID-19 and miscarriage. To identify all potentially relevant studies and articles, an extensive literature search was performed on different databases, including PubMed, Google Scholar, Ovid, Science Direct, Scopus, and Cochrane library. The following search terms (keywords) were applied in the search formula: “Severe Acute Respiratory Syndrome Corona Virus-2 (SARS-CoV-2)”, “COVID 19”, “Miscarriage”, “Pregnancy Loss”, “Repeated Miscarriages”, and “Spontaneous Abortions”. These key sentences were either used as presented, separately, or in combination with the help of the Boolean administration (OR, AND). Abstracts of the retrieved results were assessed by two independent reviewers, ΙC and AP, and full-text publications that were eligible were retrieved for further content assessment. If a study was selected by just one reviewer, the decision to include it was taken by a third reviewer (S.S.).

The literature search was conducted between 1 January 2020 and 31 August 2023. A total of 364 articles were identified from different databases. Titles and abstracts were screened by the allocated reviewers (ΙC and AP). In total, 67 full-text articles were retrieved, and 32 articles/studies were found to be eligible to provide information on this literature review. The criteria for the eligibility of the articles were that they were original articles, case reports, reviews, systematic reviews, or meta-analyses. Articles based on animal models or in a language other than English were excluded.

## 3. Miscarriage Rates and COVID-19 Infection

A retrospective study by Cavalcante et al. compared the miscarriage rate of all asymptomatic women in the first trimester (*n* = 113) with non-infected pregnant women (*n* = 172) and found that the total number of miscarriages (22.1% vs. 16.9%, *p* = 0.32) did not differ significantly. In this context, however, it is interesting to denote that miscarriage risk appears to be increased during the acute phase of COVID-19 in relation to plasma viral load, disease severity, and obstetrical risk factors [8]. In the same study, the authors conducted a meta-analysis too, using fixed and random effect models. This study revealed that the pooled percentages of miscarriages in pregnant women with COVID-19 were 15.3% and 23.1%, respectively. Therefore, it suggests that among COVID-19 cases, the miscarriage rate equates to the rate of the whole pregnant population [8].

In 1008 pregnant women with COVID-19 infection, a systematic analysis of placental pathology found chronic inflammatory pathology (including chronic villitis) in 26% of cases and increased perivillous fibrin in 33% of cases. The clinical effects of chronic inflammation were not defined in the same study as pregnancy outcomes and were not included [13]. The hospital mortality rate, preterm birth, preeclampsia, placental abruption, and disseminated intra-vascular coagulopathy (DIC) were significantly higher in women with COVID-19, and the highest rates were observed in women who tested positive for COVID-19 within 30 days of admission [14]. However, large cohort studies and meta-analyses [2,15,16,17] have demonstrated a substantial association between COVID-19 infection during pregnancy and higher rates of preterm delivery, stillbirth, and perinatal and neonatal morbidity, as well as mortality [13].

La Cour Freiesleben et al. researched the potential indicators of maternal COVID-19 infection in first trimester pregnancies and found no increase in the risk of miscarriage [18]. These results were further confirmed by Batiha et al. [9] and Rotshenker-Olshinka et al. [19]. In a single case involving a second trimester miscarriage in a woman with symptomatic coronavirus disease, swabs taken from the mouth, axillae, meconium, and fetal blood at birth all tested negative for bacterial infection and SARS-CoV-2 in the stillborn baby. The prenatal autopsy revealed no abnormalities, and SARS-CoV-2 was not detected in the fetal lung, liver, or thymus samples. Placental histology revealed unspecifically elevated intervillous fibrin deposition and mixed inflammatory infiltrates made up of neutrophils and monocytes in the subchorial region [20,21]. In another study that examined 16 placentas from patients with SARS-CoV-2, there was a higher incidence of maternal vascular malperfusion (MVM), with decidual arteriopathy—which includes atherosis, fibrinoid necrosis, and mural hypertrophy of membrane arterioles—being the most common feature [22]. Although just one patient in the current study had hypertension, maternal hypertensive diseases, such as prenatal hypertension and preeclampsia, are the main risk factors for MVM [23].

According to a study by Khosa et al., their reported cases (*n* = 76) with COVID-19 either miscarried in the first trimester or proceeded through the gestation with intrauterine growth restriction (IUGR) being evident in the second and third trimesters [24]. The results from a UK meta-analysis (*n* = 3545 women) support the hypothesis that the early-trimester pregnancy loss rate is affected by COVID-19 infection, and pregnant women with COVID-19 have a higher risk of miscarriage [25].

In research by Rashidi et al., five miscarriages were documented in women undergoing ART, and in particular ICSI, who were diagnosed with COVID-19 but without any relevant symptoms. Consequently, no association between COVID-19 and abortion was found [26]. Mothers who tested positive for COVID-19 had a spontaneous abortion rate of 2.5%. A few case studies have suggested that late second trimester miscarriages could be a complication of COVID-19 infection [26]. In another meta-analysis of 22 papers, 141 miscarriages in 8591 confirmed COVID-19 cases were examined. According to the study, 3.9% of COVID-19-infected women experienced an abortion [7].

Regarding blood groups and miscarriage rates, evidence to date shows that people in the O blood group have a reduced risk of acquiring COVID-19, whereas people in the A blood group are more likely to acquire the disease and develop a more severe form of the disease [27]. It is interesting to note that most women in this study who miscarried had an A Rh (+) blood group but without any statical correlation. Additionally, among COVID-19 patients, patients with A blood groups experienced more severe histopathological symptoms, whereas patients with O blood groups and other blood groups had less frequent symptoms. The authors suggested that COVID-19 was implicated in early pregnancy abortions by designating a more susceptible population based on blood group since patients with COVID-19 and the A Rh (+) blood group had a higher risk of pregnancy abortion than persons with other blood types [27,28]. Table 1 summarizes the outcomes of the included studies.

## 4. Discussion

Human endometrial stromal cells exhibit elevated levels of ACE2 and cellular transmembrane serine protease 2 (TMPRSS2) expression during the secretory phase, which is necessary for decidualization of these cells. For this process, human endometrial stromal cells must express high levels of ACE2 and TMPRSS2 during the secretory phase. The human placental renin–angiotensin system (RAS), which is active in the first trimester and important in both placental development and endometrial neovascularization during the peri-implantation period, includes an essential component known as ACE2 [8]. Syncytiotrophoblast, cytotrophoblast, endothelium, and vascular smooth muscles of the main and secondary maternal villi have been shown to contain ACE2. Based on the timepoint of the cycle, ACE2 expression in the endometrium varies over the course of the menstrual cycle. In the proliferative phase, epithelial cells exhibit the highest levels of ACE2 expression, whereas in the secretory phase, both stromal and epithelial cells exhibit substantial levels of ACE2 expression. The presence of ACE2 receptors at the endometrium, which could adversely affect embryo implantation, provides a plausible explanation for the spontaneous abortions observed in people with COVID-19 infection [34]. Thus, it is possible to propose numerous examples of potential impacts of COVID-19 on female reproduction: (i) COVID-19 affects the epithelial cell function in the endometrium and interferes with early embryo implantation and (ii) can affect ovarian tissue potency and granulosa cell function to reduce oocyte quality, leading to female infertility or miscarriage [1].

**Figure 1:**
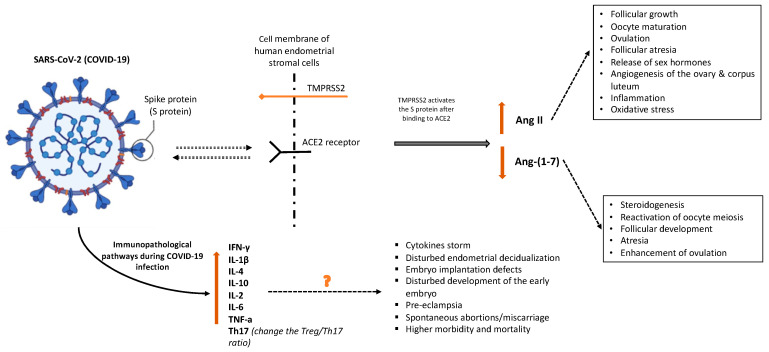
Potential pathways implicated in miscarriages associated with COVID-19 infection. Created with BioRender.

According to Yan et al., ACE2 modulates the levels of angiotensin 2 (Ang II) and angiotensin (1–7) [Ang-(1–7)]. The essential enzyme in the axis that works at aiming to balance the levels of Ang II and Ang-(1–7) is called ACE2, while it hydrolyzes Ang II into Ang (1–7) [30]. Angiotensin II controls follicular growth, oocyte maturation, and ovulation. Its function is primarily located in granulosa cells by contributing to follicular atresia, the release of sex hormones, and angiogenesis of the ovary and corpus luteum [12]. Pregnancy-related hypertension, preeclampsia, and eclampsia may be associated with the abnormal expression of Ang II, ACE2, and Ang-(1–7) since these factors primarily regulate blood pressure and fetal development throughout pregnancy [30]. COVID-19 infection in the early pregnancy and preconception period, via ACE2 activation, increases the risk for miscarriage. Reproductive failure at the immunopathological levels might result from thromboembolic events, pro-inflammatory maternal immunological responses, or direct viral activity in the uterine environment [36]. Figure 1 illustrates the potential pathways implicated in miscarriages associated with COVID-19 infection.

Angiotensin-1–7, which is present in theca-interstitial cells, is involved in steroidogenesis, the reactivation of oocyte meiosis, follicular development, atresia, and the enhancement of ovulation. The normal ovarian physiology may be altered by COVID-19 downregulation of ACE2, which could affect oocyte quality and fertility by altering follicular development and oocyte maturation. As Ang II causes inflammation, it also causes an increase in oxidative stress that may affect reproductive potential [10,11]. One previously published suggestion is that COVID-19 affects granulosa cells and ovarian tissue, thus impairing ovarian function and oocyte viability and ultimately increasing the chance of infertility and miscarriage. Additionally, COVID-19 may damage endometrial cell physiology and function that may alter the environment required for early embryo implantation [3].

Immunopathological pathways indicate that thromboembolic events, caused by this virus operating directly in the uterus, or a pro-inflammatory response by the mother’s immune system can all lead to reproductive failure [8]. COVID-19 is characterized by increased interferon-γ (IFN-γ) and IL-1β, IL-4, and IL-10 production [35]. Inflammation and viral spread both have the potential to harm tissues, causing damaged cells to release cytokines. These cytokines influence gene expression because of the triggered signaling cascade. High levels of cytokines are often linked to a range of infectious disorders, including cytokine storms, which are immunological responses characterized by the production of interferons, interleukins, tumor necrosis factors, chemokines, and several other mediators [29].

In COVID-19, preferential Th1 immunity stimulation is induced, causing a noticeable increase in cytokines that persist for at least two weeks following the initial infection. It is interesting to note that in COVID-19 cases, higher IL-6 levels have been linked to higher mortality. In experimental knockout animal models, pro-inflammatory Th-1 cytokines are frequently implicated when embryo implantation defects arise. The effects of anti-inflammatory mediators such as IL-4, IL-10, IL-13, and TGF-β are usually neutralized by pro-inflammatory cytokines such as IFN-γ, IL-1, IL-1, IL-6, and IL-12. While this type of permissive immunotolerance is associated with heightened vulnerability of the mother to infections, during pregnancy, this equilibrium is rebalanced as a potential protective effect for the fetus [35].

During COVID-19 infection, tumor necrosis factor (TNF) plasma concentration elevates, which may cause morbidity or even death from multiple organ failure. Increased amounts of TNF-a may be harmful to the development of the early embryo, according to some theories. Patients with COVID-19 infection had higher levels of inflammatory cytokines such as TNF-α, IFN-γ, IL-2, and IL-6 [1,32].

The NFKB, MAPK, PI3K, and JAK/STAT pathways are all activated by this rise in cytokines, along with increases in TNF-, IL-6, and IL-17. These signaling pathways activate and decrease the amounts of Progesterone-Induced Blocking Factor (PIBF) that lymphocytes express during pregnancy in the urine and serum. Consequently, NK cells produce TNF and target trophoblast cells to cause spontaneous abortion during pregnancy. During a typical pregnancy, lymphocytes produce progesterone and PIBF, which enhance fetal immunity, suppress NK cell activity, and reduce the chance of miscarriage by raising the production of anti-inflammatory cytokines. However, this ability is lost when the immune system becomes abnormal because of virus spread, and NK cell spread is linked to an increase in spontaneous abortions. Thus, we may assume that endometrial decidualization is disturbed in COVID-19-infected pregnant women due to the imbalance of hormones, cytokines, and pregnancy-related factors; consequently, abortion may occur [29].

Due to the increase in the ratio of Th17/Treg cells, IL-2 has also been linked to preeclampsia, miscarriage, and the IL-7/IL-7R signaling pathway in fetal miscarriage. The intricate mechanism that makes up the immune system includes Τregs and Th17 cells. Th17 and Treg cells differentiate from naive CD4+ T cells through the mediation of TGF-β. However, in the presence of TGF-β, IL-6, or IL-21, naive CD4+ T cells can differentiate into Th17 cells. A healthy pregnancy causes a shift in the Treg/Th17 ratio in favor of Treg cells. A severe COVID-19 infection may raise Th17 cells and change the Treg/Th17 ratio, leading to uncontrolled systemic inflammation that may affect pregnancy outcomes [31,33].

These types of events fit the COVID-19 “catastrophic error” theory. This model states that the viability of a pregnancy is adversely affected by the viral infection shortly after implantation or during early gestation. Given that cytokines regulate embryo orientation, apposition, docking, and invasion, an excess of pro- or anti-inflammatory signaling can have a negative effect on the pregnancy’s outcome. Measured trophoblast penetration into the decidua is dependent on pro- and anti-inflammatory inputs until the placenta is fully established; however, the excess cytokine generated by COVID-19 likely disrupts this process [9]. Additionally systemic inflammation that prevents trophoblast penetration may be the cause of miscarriage and pregnancy loss during COVID-19 infections [33].

It is important to note that apart from large studies and meta-analyses, the majority of the studies carried out to date have been small cohort studies or case series studies with a small sample size and insufficient analysis to determine the correlation between the clinical features of pregnant women with COVID-19 and the risk of miscarriage, or to analyze the risk of miscarriage in pregnant women infected with COVID-19 [8].

## 5. Conclusions

COVID-19 is a modern worldwide pandemic that affected and continues to affect millions of people around the world. Since infections are persisting, the correlation of the infection with various health manifestations such as miscarriages should be determined to provide improved medical management.

Given that ACE2 controls the development of follicles and ovulation, normalizes angiogenesis and luteal degeneration, and may alter the dynamics of embryonic development and endometrial tissue, COVID-19 has been proposed as a significant candidate in exerting a severe impact on female reproductive potential, and especially during the course of pregnancy. The presence of ACE2 receptors at the endometrium, which could adversely affect embryo implantation, provides a plausible explanation for the spontaneous abortions observed in people with COVID-19 infection.

To ascertain whether miscarriages and COVID-19 infection are related, this review combined the reported outcomes from previously published studies involving pregnant COVID-19-infected women to provide an overview of this potential relationship. Overall, these findings suggest that COVID-19 may result in miscarriage, preterm labor, and fetal distress. The miscarriage rate among COVID-19 cases is within the usual range for the general pregnant population, according to most meta-analyses and cohort studies. To provide a conclusive and evidence-based opinion on this potential association, larger, more controlled, and more homogeneous studies are necessary, considering the potential consequences of COVID-19 infection in the reproductive system.

Additionally, a more detailed and systematic approach should focus on cellular and molecular mechanisms related to COVID-19 infection and pregnancy outcomes. A worldwide data repository could be a major utility as a global data poll, and on this basis, the European Network of Centers of Pharmacoepidemiology and Pharmacovigilance (ENCENP) created the COVI-PREG registry, which aims to collect data to understand the natural history of COVID-19 among pregnant women and the impact on maternity, pregnancy and neonatal outcomes (encenp.eu). Such initiatives should be further supported and extended.

## Figures and Tables

**Table 1:** Studies included in the review.

Study	Year	Study Design	Main Outcome
Afrooz N et al. [28]	2022	Review	Immune system suppression disrupts the pregnancy process by affecting the profiles of cytokines, coagulation systems (D-dimer), and hormones
Aho Glele L.S. et al. [15]	2022	Meta-analysis	COVID-19 infection is associated with preterm birth, and may be associated with preeclampsia
Alberca R.W. et al. [20]	2020	Review	A single case involving a second trimester miscarriage
Allotey J et al. [2]	2020	Systematic review and meta-analysis/435 studies	Pregnant women with COVID-19 have increased risk for preterm delivery, maternal death, and ICU admission.
Arican C.D. et al. [27]	2022	Article	Significant vascular changes in the placentas of infected pregnant women
Balachandren N. et al. [25]	2022	Prospective cohort study/3041 pregnancies	COVID-19 infection in the first trimester may have a higher risk of miscarriage
Batiha O. et al. [9]	2020	Review	Miscarriages were only reported in SARS infections. Preterm birth, preeclampsia, and caesarean delivery were more common in COVID-19-infected mothers
Baud D. et al. [21]	2020	Case report	One case of miscarriage during the second trimester of pregnancy related to placental infection
Bilal M.Y. et al. [29]	2021	Article	Infection by COVID-19 can lead to miscarriage, respiratory distress, and preterm delivery
Carneiro Gomes P.R. et al. [12]	2021	Review	Infection in the primordial germ cells causes a malfunction of the reproductive glands and a possible alteration in the gametes
Cavalcante M.B. et al. [8]	2021	Systematic review (17 studies)/meta-analysis (10 studies—223 cases)	Miscarriage rates in pregnant women with COVID-19 were 15.3% (95% CI 10.94–20.59) and 23.1% (95% CI 13.17–34.95) using fixed and random effect models, respectively
Cornish E.F. et al. [13]	2022	Review	A systematic analysis of placental pathology in 1008 pregnant women with COVID-19 infection found chronic inflammatory pathology (including chronic villitis) in 26% of cases and increased perivillous fibrin in 33% of cases
Hajialiakbari N, S.D. et al. [7]	2022	Meta-analysis (22 studies/8591 infected pregnant women, 141 abortions)	Miscarriage incidence was 3.9% (95% CI 0.023–0.063) in infected women with COVID-19
Jing Y. et al. [30]	2020	Review	Pregnancy-related hypertension, preeclampsia, and eclampsia may be associated with the abnormal expression of Ang II, ACE2, and Ang-(1–7) in COVID-19 infection
Kazami F.N. et al. [16]	2021	Systematic Review	Increased risk of miscarriage in COVID-19-positive women Placental inflammation may result in fetal growth retardation and induce abortion
Khosa S.N. et al. [24]	2021	Case series/76 women	Of 76 pregnant women, 41 (54%) had a miscarriage with positive COVID-19 tests and 24 (31.6%) had spouses who had COVID-19 positive tests
La Cour Freiesleben N. et al. [18]	2021	Cohort study/1019 women	Maternal COVID-19 infection in first trimester does not increase the risk of miscarriage
Lee W.Y. et al. [11]	2021	Review	SARS-CoV-2 infection, via ACE2 receptor, may disrupt ovarian function and oocyte quality
Li R. et al. [1]	2020	Review	COVID-19 affects the epithelial cell function in the endometrium and interferes with early embryo implantation COVID-19 can affect ovarian tissue potency and granulosa cell function and reduce oocyte quality
Litman E.A. et al. [14]	2022	Cohort study	There was no significant difference in the prevalence of stillbirths between women with and without COVID-19 (0.6% vs. 0.5%)
Liu C. et al. [10]	2021	Review	COVID-19 can affect the follicular membrane and granulosa cells of the ovary, reduce the quality of oocytes, and lead to miscarriages
Muyayalo K.P. et al. [31]	2020	Review	The increased IL-7/IL-7R signaling pathway has been associated with fetal miscarriage
Nateghi R. et al. [32]	2021	Review of literature	The higher levels of inflammatory cytokines such as TNF-α, IFN-γ, IL-2, and IL-6 in COVID-19 disease may affect early embryo development
Rashidi B.H. et al. [26]	2022	Case series/38 women	No correlation in neonatal morbidity during pregnancy in pregnancies with COVID-19 infection
Rotshenker-Olshinka K. et al. [19]	2021	Cohort study/285 women	No correlation in first trimester miscarriages and ongoing pregnancies
Saadedine M. et al. [33]	2023	Review	A severe COVID-19 infection may raise Th17 cells and change the Treg/Th17 ratio, leading to uncontrolled systemic inflammation that affects pregnancy outcomes
Sandulescu M.S. et al. [34]	2022	Review	COVID-19 infection interferes with ACE2 receptors found at the endometrium and negatively affects embryo implantation and/or fetal distress in pregnant patients
Shanes E.D. et al. [22]	2020	Article	Higher incidence of maternal vascular malperfusion (MVM), with decidual arteriopathy in COVID-19 pregnancies
Sharma I. et al. [3]	2021	Review	COVID-19 affects granulosa cells and ovarian tissue and increases the risk of miscarriage and infertility
Sills E.S. et al. [35]	2020	Review	In COVID-19, preferential Th1 immunity stimulation is induced and may affect embryo implantation
Vesce F. et al. [36]	2022	Review	Miscarriages in COVID-19 patients, at immunopathological levels, might result from thromboembolic events, pro-inflammatory maternal immunological responses, or direct viral activity in the uterine environment
Villar J. et al. [17]	2021	Cohort study/2130 women	COVID-19 infection in pregnancy was associated with increased risk for neonatal complications

## Data Availability

Not applicable.

## References

[1] Li R., Yin T., Fang F., Li Q., Chen J., Wang Y., Hao Y., Wu G., Duan P., Wang Y. (2020). Potential risks of SARS-CoV-2 infection on reproductive health. Reprod. Biomed. Online.

[2] Allotey J., Stallings E., Bonet M., Yap M., Chatterjee S., Kew T., Debenham L., Llavall A.C., Dixit A., Zhou D. (2020). Clinical manifestations, risk factors, and maternal and perinatal outcomes of coronavirus disease 2019 in pregnancy: Living systematic review and meta-analysis. BMJ.

[3] Sharma I., Kumari P., Sharma A., Saha S.C. (2021). SARS-CoV-2 and the reproductive system: Known and the unknown!!. Middle East Fertil. Soc. J..

[4] Kanmaz A.G., Inan A.H., Beyan E., Budak A. (2019). The effects of threatened abortions on pregnancy outcomes. Ginekol. Pol..

[5] Zinaman M.J., Clegg E.D., Brown C.C., O’Connor J., Selevan S.G. (1996). Estimates of human fertility and pregnancy loss. Fertil. Steril..

[6] Wilcox A.J., Weinberg C.R., O’Connor J.F., Baird D.D., Schlatterer J.P., Canfield R.E., Armstrong E.G., Nisula B.C. (1988). Incidence of early loss of pregnancy. N. Engl. J. Med..

[7] Hajialiakbari N., Schwartz D., Javaheri A., Karimi-Zarchi M., Golshan-Tafti M., Dastgheib S.A., Bahrami R., Zanbagh L., Neamatzadeh H. (2022). A Meta-Analysis for Frequency of Miscarriage in Pregnant Women with COVID-19. World J. Peri Neonatol..

[8] Cavalcante M.B., de Melo Bezerra Cavalcante C.T., Cavalcante A.N.M., Sarno M., Barini R., Kwak-Kim J. (2021). COVID-19 and miscarriage: From immunopathological mechanisms to actual clinical evidence. J. Reprod. Immunol..

[9] Batiha O., Al-Deeb T., Al-Zoubi E., Alsharu E. (2020). Impact of COVID-19 and other viruses on reproductive health. Andrologia.

[10] Liu C., Mu C., Zhang Q., Yang X., Yan H., Jiao H. (2021). Effects of Infection with SARS-CoV-2 on the Male and Female Reproductive Systems: A Review. Med. Sci. Monit..

[11] Lee W.Y., Mok A., Chung J.P.W. (2021). Potential effects of COVID-19 on reproductive systems and fertility; assisted reproductive technology guidelines and considerations: A review. Hong Kong Med. J..

[12] Carneiro Gomes P.R., Rodrigues da Rocha M.D., da Rocha Coelho F.A., Sousa Pinho de Lira J.A., de Sousa Carmo R.R., Silva Nascimento H.M., Marques de Oliveira S., Rodrigues da Silva W., Galdino Medeiros R., Pereira Alves E.H. (2021). Alterations of the male and female reproductive systems induced by COVID-19. Wien. Klin. Wochenschr..

[13] Cornish E.F., McDonnell T., Williams D.J. (2022). Chronic Inflammatory Placental Disorders Associated With Recurrent Adverse Pregnancy Outcome. Front. Immunol..

[14] Litman E.A., Yin Y., Nelson S.J., Capbarat E., Kerchner D., Ahmadzia H.K. (2022). Adverse perinatal outcomes in a large United States birth cohort during the COVID-19 pandemic. Am. J. Obstet. Gynecol. MFM.

[15] Aho Glele L.S., Simon E., Bouit C., Serrand M., Filipuzzi L., Astruc K., Kadhel P., Sagot P. (2022). Association between SARS-CoV-2 infection during pregnancy and adverse pregnancy outcomes: A re-analysis of the data reported by Wei et al. (2021). Infect. Dis. Now.

[16] Kazemi S.N., Hajikhani B., Didar H., Hosseini S.S., Haddadi S., Khalili F., Mirsaeidi M., Nasiri M.J. (2021). COVID-19 and cause of pregnancy loss during the pandemic: A systematic review. PLoS ONE.

[17] Villar J., Ariff S., Gunier R.B., Thiruvengadam R., Rauch S., Kholin A., Roggero P., Prefumo F., do Vale M.S., Cardona-Perez J.A. (2021). Maternal and Neonatal Morbidity and Mortality Among Pregnant Women With and Without COVID-19 Infection: The INTERCOVID Multinational Cohort Study. JAMA Pediatr..

[18] La Cour Freiesleben N., Egerup P., Hviid K.V.R., Severinsen E.R., Kolte A.M., Westergaard D., Fich Olsen L., Praetorius L., Zedeler A., Christiansen A.H. (2021). SARS-CoV-2 in first trimester pregnancy: A cohort study. Hum. Reprod..

[19] Rotshenker-Olshinka K., Volodarsky-Perel A., Steiner N., Rubenfeld E., Dahan M.H. (2021). COVID-19 pandemic effect on early pregnancy: Are miscarriage rates altered, in asymptomatic women?. Arch. Gynecol. Obstet..

[20] Alberca R.W., Pereira N.Z., Oliveira L., Gozzi-Silva S.C., Sato M.N. (2020). Pregnancy, Viral Infection, and COVID-19. Front. Immunol..

[21] Baud D., Greub G., Favre G., Gengler C., Jaton K., Dubruc E., Pomar L. (2020). Second-Trimester Miscarriage in a Pregnant Woman With SARS-CoV-2 Infection. JAMA.

[22] Shanes E.D., Mithal L.B., Otero S., Azad H.A., Miller E.S., Goldstein J.A. (2020). Placental Pathology in COVID-19. Am. J. Clin. Pathol..

[23] Weiner E., Feldstein O., Tamayev L., Grinstein E., Barber E., Bar J., Schreiber L., Kovo M. (2018). Placental histopathological lesions in correlation with neonatal outcome in preeclampsia with and without severe features. Pregnancy Hypertens..

[24] Khosa F., Naeem M., Sultan Z., Rizwan A.S., Sher S.J., Ali N. (2021). Impacts of COVID-19 Pandemic on the Early Trimester Pregnancies. Pak. J. Med. Health Sci..

[25] Balachandren N., Davies M.C., Hall J.A., Stephenson J.M., David A.L., Barrett G., O’Neill H.C., Ploubidis G.B., Yasmin E., Mavrelos D. (2022). SARS-CoV-2 infection in the first trimester and the risk of early miscarriage: A UK population-based prospective cohort study of 3041 pregnancies conceived during the pandemic. Hum. Reprod..

[26] Rashidi B.H., Bandarian F., Bandarian M. (2022). Maternal and neonatal outcomes of pregnancies of infertile women during the COVID-19 pandemic: A real world evidence. JBRA Assist. Reprod..

[27] Arican C.D. (2022). The Relationship between the COVID-19 Pandemic and Early Pregnancy Abortions. Eur. J. Med. Health Sci..

[28] Afrooz N., Mahmoudi S.K., Yazdizadeh M., Jahanshahiafshar Z., Sabernia N., Rohaninasab M. (2022). The evaluation of COVID-19 effect on pregnancy loss; a molecular and diagnostic approach. Immunopathol. Persa.

[29] Bilal M.Y., Katara G., Dambaeva S., Kwak-Kim J., Gilman-Sachs A., Beaman K.D. (2021). Clinical molecular genetics evaluation in women with reproductive failures. Am. J. Reprod. Immunol..

[30] Jing Y., Run-Qian L., Hao-Ran W., Hao-Ran C., Ya-Bin L., Yang G., Fei C. (2020). Potential influence of COVID-19/ACE2 on the female reproductive system. Mol. Hum. Reprod..

[31] Muyayalo K.P., Huang D.H., Zhao S.J., Xie T., Mor G., Liao A.H. (2020). COVID-19 and Treg/Th17 imbalance: Potential relationship to pregnancy outcomes. Am. J. Reprod. Immunol..

[32] Nateghi R., Ghashghaei S.H., Shokoohian B., Hezavehei M., Ebrahimi B., Shahverdi A.H., Mashayekhi M., Shpichka A., Timashev P., Nasr-Esfahani M.H. (2021). Female Reproductive Health in SARS-CoV-2 Pandemic Era. Int. J. Fertil. Steril..

[33] Saadedine M., El Sabeh M., Borahay M.A., Daoud G. (2023). The influence of COVID-19 infection-associated immune response on the female reproductive systemdagger. Biol. Reprod..

[34] Sandulescu M.S., Vaduva C.C., Siminel M.A., Dijmarescu A.L., Vrabie S.C., Camen I.V., Tache D.E., Neamtu S.D., Nagy R.D., Carp-Veliscu A. (2022). Impact of COVID-19 on fertility and assisted reproductive technology (ART): A systematic review. Rom. J. Morphol. Embryol..

[35] Sills E.S., Wood S.H. (2020). An Experimental Model for Peri-conceptual COVID-19 Pregnancy Loss and Proposed Interventions to Optimize Outcomes. Int. J. Mol. Cell. Med..

[36] Vesce F., Battisti C., Crudo M. (2022). The Inflammatory Cytokine Imbalance for Miscarriage, Pregnancy Loss and COVID-19 Pneumonia. Front. Immunol..

